# Robo1 Forms a Compact Dimer-of-Dimers Assembly

**DOI:** 10.1016/j.str.2017.12.003

**Published:** 2018-02-06

**Authors:** Nataliia Aleksandrova, Irina Gutsche, Eaazhisai Kandiah, Sergiy V. Avilov, Maxim V. Petoukhov, Elena Seiradake, Andrew A. McCarthy

**Affiliations:** 1 European Molecular Biology Laboratory, Grenoble Outstation, 71 avenue des Martyrs, 38042 Grenoble, France; 2 University Grenoble Alpes, CNRS, CEA, IBS, 71 avenue des Martyrs, 38044 Grenoble, France; 3 European Molecular Biology Laboratory, Hamburg Unit, Notkestrasse 85, Hamburg 22607, Germany; 4 Federal Scientific Research Centre “Crystallography and Photonics” of Russian Academy of Sciences, Leninsky Prospect 59, 119333 Moscow, Russian Federation; 5 A. N. Frumkin Institute of Physical Chemistry and Electrochemistry RAS, Leninsky Prospect 31, 119071 Moscow, Russian Federation; 6 N.N. Semenov Institute of Chemical Physics of Russian Academy of Sciences, Kosygina Street 4, 119991 Moscow, Russian Federation; 7 Department of Biochemistry, University of Oxford, South Parks Road, OX1 3QU Oxford, UK

**Keywords:** robo, slit, receptor, X-ray crystallography, electron microscopy

## Abstract

Roundabout (Robo) receptors provide an essential repulsive cue in neuronal development following Slit ligand binding. This important signaling pathway can also be hijacked in numerous cancers, making Slit-Robo an attractive therapeutic target. However, little is known about how Slit binding mediates Robo activation. Here we present the crystal structure of Robo1 Ig1-4 and Robo1 Ig5, together with a negative stain electron microscopy reconstruction of the Robo1 ectodomain. These results show how the Robo1 ectodomain is arranged as compact dimers, mainly mediated by the central Ig domains, which can further interact in a “back-to-back” fashion to generate a tetrameric assembly. We also observed no change in Robo1 oligomerization upon interaction with the dimeric Slit2-N ligand using fluorescent imaging. Taken together with previous studies we propose that Slit2-N binding results in a conformational change of Robo1 to trigger cell signaling.

## Highlights

• Robo1 Ig1-Ig4 adopts an extended conformation
• Robo1 ectodomain forms a compact tetrameric assembly *in vitro*
• No change in Robo1 oligomerization occurs upon interaction with Slit2-N

## Graphical Abstract

**Figure:**
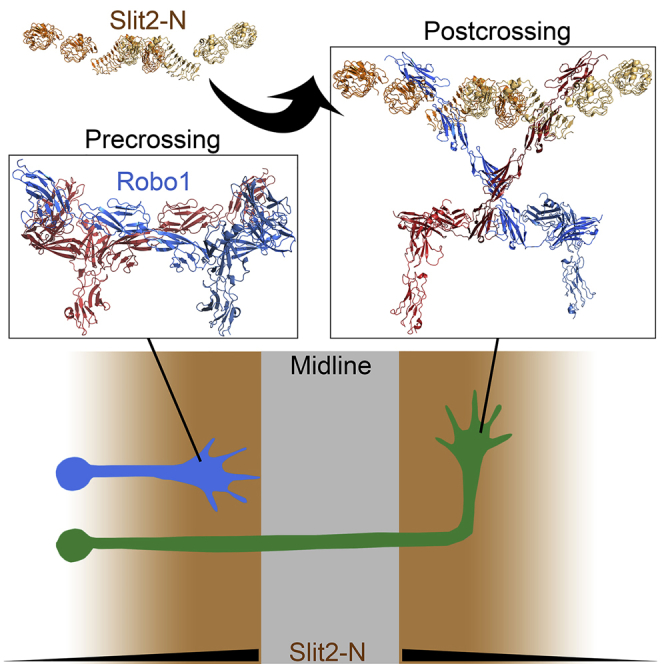

## Teaser

Slit-Robo signaling is an essential neuronal development pathway, but little is known about how Slit binding to Robo is transmitted across the cell membrane. Aleksandrova et al. present a structural model of a Robo ectodomain to provide insight into receptor activation upon Slit binding.

## Introduction

During bilateral CNS development the commissural neurons must cross the midline once, ensuring the proper connection of both sides, in a process that is dependent on coordinated attractive and repulsive cues (Garbe and Bashaw, 2004). Roundabout (Robo) receptors provide a critical repulsive cue upon binding Slit, a protein secreted by the midline glial cells, and prevent the re-crossing of Robo-expressing neurons (Dickson and Gilestro, 2006). More recently, Slit-Robo signaling has been observed to play an important role in a variety of processes outside the CNS system: by acting as a potent inhibitor of platelet formation (Patel et al., 2012); regulating cancer cell migration and proliferation (Ballard and Hinck, 2012); inhibiting the migration of immature dendritic cells during HIV-1 infection (Prasad et al., 2012); and reducing the loss of intestinal stem cells during chemoradiotherapy (Zhou et al., 2013).

**Figure 1:**
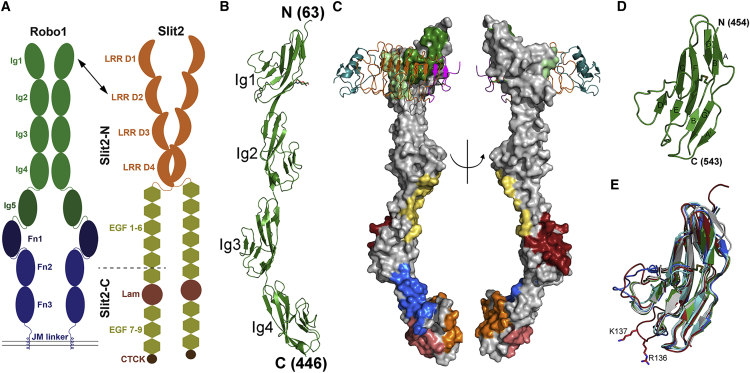
Structure of Human Robo1 Ig1-4 and Ig5 (A) The domain composition of Robo1 and Slit2. The Robo1 Ig and Fn domains are colored green and blue, respectively. The Slit2 LRR, EGF, Lamin, and CTCK domains are colored orange, yellow, red, and brown, respectively. The cleave site of Slit2 is indicated by a dashed line. (B) Robo1 Ig1-4 domains adopt an extended structure. The N-glycosylation at N160 is shown in stick representation. (C) The major crystallographic contacts are mediated by Ig1, Ig3, and Ig4. Interface 1 is symmetric and mediated by Ig4 (blue); interface two is mediated by Ig2-3 (yellow) and Ig4 (orange); and interface three is mediated by Ig3 (red) and Ig4 (salmon); interface four is mediated by Ig1 (light and dark green) and overlaps the Slit2 D2 binding site, illustrated as a ribbon representation (N- and C-terminal caps colored in magenta and cyan, respectively, and LRR colored in orange). (D) The Robo1 Ig5 domain structure showing a canonical I-set fold. (E) A superposition of Robo1 Ig domains with Ig1, Ig2, Ig3, Ig4, and Ig5 colored in red, gray, cyan, blue and green, respectively. One potential conformation of K137 and R136 (disordered) is shown in stick representation to highlight the Robo1 Ig1 heparin binding region (βE-βF loop).

Three Robo receptors (Robo, Robo2, and Robo3) have been characterized in *Drosophila* and four (Robo1-4) have been identified in vertebrates (Chédotal, 2007, Dickson and Gilestro, 2006). The *Drosophila* and vertebrate Robo1-3 are most similar, containing five immunoglobulin (Ig) and three fibronectin (Fn) domains in their extracellular region (Figure 1A). Robo4 is a smaller endothelial and vascular specific receptor (Huminiecki et al., 2002), having only two Ig and Fn domains. These extracellular domains are followed by a membrane proximal region, a single transmembrane helix, and an unstructured intracellular region containing conserved sequence motifs used to mediate the binding of effector proteins (Chédotal, 2007). The crystal structures of several extracellular domains of Robo1 have been determined, these include the Ig1-2 region harboring the Slit2 ligand binding region on Ig1 (Fukuhara et al., 2008, Liu et al., 2004, Morlot et al., 2007), and the juxtamembrane region spanning Fn2-3 (Barak et al., 2014).

Slits act as repulsive cues in both vertebrates and invertebrates upon binding Robo receptors (Brose et al., 1999, Ypsilanti et al., 2010). Slit was discovered in *Drosophila* and several homologs, Slit1-3, were subsequently identified in mammals (Dickson and Gilestro, 2006). Slits are large secreted glycoproteins containing four leucine-rich repeat (LRR) domains (D1-D4), several epidermal growth factor (EGF) repeats, a laminin-G domain, and a C-terminal cysteine knot (Dickson and Gilestro, 2006) (Figure 1A). Slits can be cleaved by an unidentified process (Nguyen Ba-Charvet et al., 2001, Wang et al., 1999) to produce an N-terminal fragment (Slit-N) harboring the Robo binding site within LRR D2 (Howitt et al., 2004) with a well-characterized repulsive activity.

Although Slit-Robo signaling has been intensely studied there is still a clear lack of knowledge on how exactly their interaction is relayed across the membrane. While Slit D1-4 was reported to be monomeric in solution (Hohenester, 2008), it was shown that Slit2 dimerization is mediated by the LRR D4 domain (Howitt et al., 2004, Seiradake et al., 2009) (Figure 1A). This suggested that a reorganization of the Robo receptor oligomeric state upon Slit binding was required for intracellular signaling to occur (Hohenester, 2008). Recent advanced light microscopy studies contradict this model, illustrating that the oligomerization state of Robo1 does not change, regardless of whether Slit2-N is present or not (Zakrys et al., 2014). Previous analysis showed that the ectodomain (ECD) is mainly responsible for Robo1-Robo1 interactions (Hivert et al., 2002, Liu et al., 2004), and further studies indicate that this was largely mediated by the Ig domains *in vivo* (Zakrys et al., 2014). Several studies have observed that both *Drosophila* Robo1 and mammalian Robo1 can undergo cleavage by matrix metalloproteases, resulting in receptor shedding and subsequent downstream signaling (Coleman et al., 2010, Seki et al., 2010). While this mechanism is supported by structural and biochemical studies on the Robo1 juxtamembrane (Barak et al., 2014), it has not been conclusively shown to be Slit dependent. Moreover, recent *Drosophila* genetic experiments, supported by *in vivo* data, have shown that endocytosis of the Robo receptor in response to Slit binding is necessary for repulsive signaling at the midline (Chance and Bashaw, 2015).

To gain a more detailed understanding of a Robo1 dimer interaction we undertook an integrated structural study of several Robo1 constructs, which was complemented by light microscopy experiments. Our negative stain electron microscopy (EM) results indicate that the Robo1 ECD folds back on itself in a looping configuration, thereby forming a larger tetrameric structural arrangement consisting of a “dimer-of-dimers” in a putative inactive conformation. These results support the idea that the whole Robo1 ECD is required for oligomerization, and that the Ig domain region is largely responsible for this (Zakrys et al., 2014). We confirmed that an oligomeric arrangement, which persists on addition Slit2-N, is also observed on the cell surface. These results are consistent with a mechanistic model in which a Slit2-N-induced conformational change of Robo1 is required for receptor activation.

## Results

### Robo1 Oligomerization

Robo1 was previously shown to exist as monomers, dimers, and oligomers (Barak et al., 2014, Liu et al., 2004, Zakrys et al., 2014). To determine the principal species we produced Robo1 ECD constructs (S1A). Using size-exclusion chromatography we routinely observed that Robo1 ECD exists in different oligomeric states (Figure S1B). The majority of Robo1 is tetrameric, but a significant portion of dimers were also observed. These results were confirmed by multiangle light scattering (MALS), in which a range of oligomeric states, from 100 to 500 kDa, spanning monomers to tetramers, were observed (Figure S1C). Furthermore, gradient-fixation ultracentrifugation experiments are consistent with a major tetrameric assembly of Robo1 (Figures S1D and S1E).

### Structural Analysis of Robo1 Ig1-4 and Ig5

The Robo1 Ig1-4 domains are arranged in an extended conformation, ∼170 Å in length (Figure 1B), with essentially no polypeptide linker between any of the four domains. Two potential N-glycosylation sites at N160 and N175 were identified by sequence analysis. A close inspection of the electron density for these residues reveals clear glycosylation at N160, while N175 is most likely not glycosylated. A comparison with the two Robo1 Ig1-2 structures reported previously (Morlot et al., 2007) shows that these domains are most similar to the second conformation (PDB: 2v9r; Figure S2A). *Drosophila* Robo1 (dRobo1) Ig1-2 was also crystallized in the presence, and absence, of a heparin octasaccharide (Fukuhara et al., 2008). A relative domain rotation of 35° around I150 was observed in the heparin bound form (PDB: 2vra), which is most similar to the first Robo1 Ig1-2 conformation (PDB: 2v9q). The apo form (PDB: 2vr9) is most similar to both Robo1 Ig1-2 conformation 2 (PDB: 2v9r) and Robo1 Ig1-4 (PDB: 5o5g; Figure S2B). We obtained similar Robo1 Ig1-4 crystals in a different crystallization condition with a c axis ∼10 Å longer (Table 1). Although these diffracted to a higher resolution, the Ig1 domain was disordered. Otherwise the structures are identical, apart from the βC-βD loop region of Ig4, which is better resolved in the higher-resolution dataset. The Ig1 domain disorder observed is consistent with the Ig1-2 domain movements observed previously (Fukuhara et al., 2008, Morlot et al., 2007), and the recent observation that heparin binding can induce a minor change in the conformation, or dynamics, of the Ig2 domain in Robo1 Ig1-2 (Li et al., 2015).

**Table 1:** Diffraction Data Collection and Refinement Statistics

Crystal Parameters	Robo1 Ig5	Robo1 Ig1-4 (1)	Robo1 Ig1-4 (2)
**Data Collection**			

Wavelength (Å)	0.9393	0.9393	0.9393
Space group	P6_1_	C222_1_	C222_1_
Cell dimensions			
a, b, c (Å)	80.8, 80.8, 26.83	44.2, 99.7, 238.7	43.5, 99.1, 247.2
α, β, γ (°)	90, 90, 120	90, 90, 90	90, 90, 90
Molecules per asymmetric unit	1	1	1
Resolution (Å) (final shell)	50–3.0 (3.2–3.0)	30–3.0 (3.2–3.0)	20–2.5 (2.7–2.5)
Observed reflections	6,223	41,535 (7,197)	61,677 (4,596)
Unique reflections	2,082	10,679 (1,866)	17,779 (1,772)
Completeness (%) (final shell)	98.3 (94.1)	99.6 (98.7)	97.8 (82.7)
R_pim_ (%) (final shell)	14.8 (79.8)	13.0 (44.7)	9.5 (58.1)
<I/σ(I)> (final shell)	7.3 (1.5)	4.0 (1.4)	4.9 (1.3)

**Model Quality Indicators**			

R_cryst_ (%)	19.3	19.8	21.2
R_free_ (%)	24.5	25.8	27.0
RMSD			
Bonds (Å)	0.008	0.009	0.009
Angles (°)	1.1	1.2	1.2

RMSD, root-mean-square deviation.

We used the EPPIC server to search for possible biologically relevant interfaces. No intermolecular interactions consistent with a biological oligomeric organization were identified and confirmed by small angle X-ray scattering (SAXS) (Figure S3). Nevertheless, four crystallographic contact interfaces are observed (Figure 1C). The first results in a dimeric arrangement between 2-fold symmetric molecules along the b axis, and is primarily mediated by the Ig4 domain. This interface, which spans residues 378–394 and 429–436, has the largest buried surface (663 Å^2^ of each molecule's surface area), and is predominantly hydrophobic in nature. These results, together with high surface sequence conservation (Figure S2C), are suggestive of biological relevance. The next largest interface buries 616 Å^2^ from each molecule and is mediated by residues from Ig2 (183–186 and 228–231) and Ig3 (260–264 and 338–341) from one molecule with residues 367–371 and 400–417 from Ig4. The third interface buries 481 Å^2^ from each molecule and is largely electrostatic in nature, comprising seven H-bonds and two salt bridges. This interaction area is mediated between Ig3 (residues 292–297 and 301–311) and Ig4 (residues 357–359, 385–388, 421–427, and 439–444). The last interface, which buries 466 Å^2^ from each molecule, is mediated exclusively by residues 71–72, 86–96, and 122–126 from one Ig1 domain interacting with residues 79–84, 115–120, and 128–131 from the second Ig1. Both interaction surfaces partially overlap with the Slit2 D2 binding surface on Ig1 (Figure 1C), but Slit2 D2 binding to the first is more extensive compared with the second.

Robo1 Ig5 belongs to the I-set Ig-like domain class (Figure 1D), as do the other Robo1 domains, and is essentially identical to the nuclear magnetic resonance structure deposited in the PDB (PDB: 2eo9), with a root-mean-square deviation (RMSD_*90cα*_) of 0.9 Å. An inspection of the potential N-glycosylation site at N463 reveals no additional density. All five Robo1 Ig domains are very similar, the main conformational difference occurring in the βC-βD loop region (Figure 1E). Interestingly, the βE-βF loop region in Ig1, which contributes two sequence conserved basic residues (K137 and R136) required for heparin binding (Fukuhara et al., 2008), is longer than those observed in the other four Ig domains (Figure 1E).

### SAXS of Robo1 ECD Constructs

To confirm if Robo1 Ig1-4 is monomeric in solution, and since we were unable to crystallize Robo1 Ig1-5, we used SAXS to determine their overall shape. The experimental scattering patterns (Figure S3A) and overall structural parameters computed from the SAXS data are given in Table S1. The structural parameters obtained correspond to a monomeric state for both constructs in solution. Several independent *ab initio* runs yielded reproducible molecular shapes, and the average models generated are consistent with the Robo1 Ig1-4 structure obtained (Figure S3B). The fit directly computed from the atomic model of Robo1 Ig1-4 yields a very good agreement with the experimental SAXS profile and a χ^2^ = 1.4 (Figure S3A; Table S1). Rigid body modeling of the Robo1 Ig1-5 construct by adding the Ig5 domain determined allowed us to fit the corresponding SAXS data rather well (χ^2^ = 1.3; Figure S3A; Table S1), yielding a model similar to the previously computed *ab initio* shape (Figure S3B).

### Negative-Stain Reconstruction of Robo1 ECD

**Figure 2:**
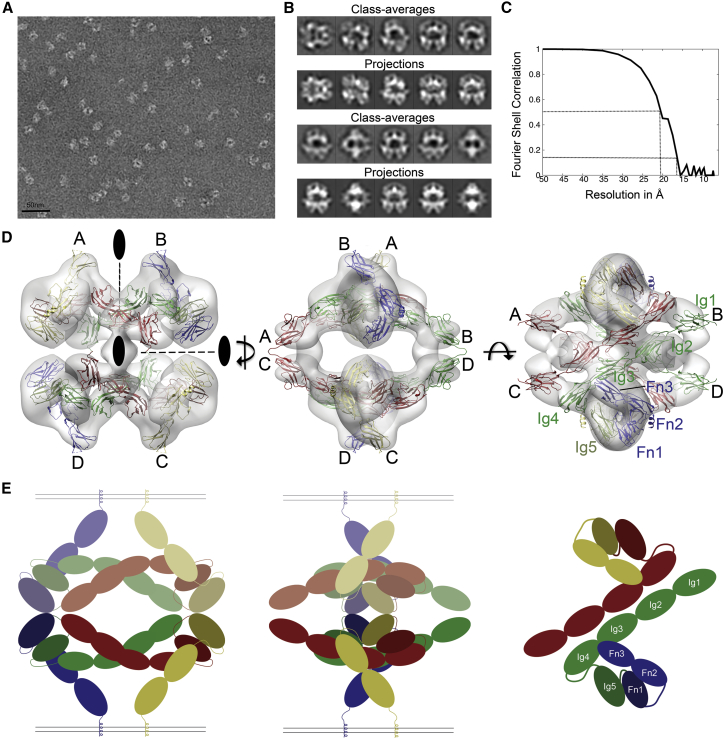
EM Reconstruction of the Robo1 ECD (A) A representative negative stain EM micrograph is shown. Scale bar, 50 nm. (B) Comparison of 2D class averages after alignment and classification with the back projections of the final refined volume. (C) Gold-standard Fourier shell correlation (FSC) curves indicating a resolution of 16 Å according to FSC = 0.143 criterion. (D) 3D reconstruction and domain assignment of Robo1 ECDs. Three orientations are shown to illustrate the “dimer-of-dimers” configuration. The monomers (A, B, C, and D) form inactive dimers (A/B and C/D), which further assemble in a “back-to-back” fashion as a tetramer. (E) Schematic overview of the major Robo1 back-to-back dimer conformation in similar orientations to (D). For illustration purposes the membrane insertion region was included, one dimer was paled, and only one dimer shown in the final orientation. The Ig and Fn domains are labeled and colored green and blue or red and yellow, respectively, for each dimer.

The yields of Robo1 ECD precluded extensive crystallization trials so we used negative-stain EM (Figure 2A) to determine the oligomeric state and obtain low-resolution structural information. The ensuing 2D class averages (Figure 2B) showcased a set of distinct well-defined shapes with clear internal structure and a notable dihedral D2 symmetry, which was also revealed in an initial 3D EM map, and therefore imposed in the subsequent 3D refinement procedure. The final 3D EM reconstruction was filtered to 20 Å (Figure 2C), has very similar overall dimensions of ∼180–190 Å in each direction, and a volume (∼1.3 × 10^6^ Å^3^) consistent with a tetrameric assembly of Robo1 ECD. The D2 symmetry allowed the identification of two independent regions in the EM map, resulting in a reduction of one dimension to ∼90 Å and a volume concomitant to Robo1 ECD dimers. The longest continuous fragment of Robo1 available, the Ig1-4 structure, is ∼170 Å long and has a D_max_ of ∼185 Å. We therefore used this information as a constraint to position Ig1-4 in what we term the “major” dimeric map volume. Several manual modeling attempts allowed us to identify only one suitable position for the Robo1 Ig1-4 fragment, resulting in an antiparallel orientation within this dimeric region, which is consistent with previous studies (Zakrys et al., 2014). The other potential models result in Ig1 protruding from the map or significant clashes occurring when the full D2 symmetry was imposed. The Robo1 Ig1-5 has a D_max_ of ∼200 Å, indicating that Robo1 Ig1-5 does not adopt an extended conformation and consistent with the EM map having a largest dimension of ∼190 Å. This constraint, including an eight-amino acid linker region between Ig4 and Ig5, provided the basis to model Ig5 in a looping back orientation. The Fn2-3 region, measuring ∼90 Å, could then be modeled in the shortest dimension of the major dimeric volume to position the two juxtamembrane regions in a membrane-spanning orientation. Lastly, the Fn1 model was oriented to provide a link between Ig5 and Fn2-3. The fitting of this model was further improved using Chimera and iMODFIT, resulting in a consensus pseudoatomic model of the Robo1 ECD (Figure 2D).

In our model, the Robo1 ECD forms a compact “dimer-of-dimers” tetrameric D2 assembly. The major dimeric interface is formed by the Ig1-4 region, which is oriented in an antiparallel manner (Figures 2D and 2E). This dimer interface is largely mediated by Ig3, with further contributions from Ig1 and Ig4. This is the same region shown to mediate the dimerization of Robo2 and direct the mediolateral positioning of axons in the CNS of *Drosophila* (Evans and Bashaw, 2010). The minor dimeric interface responsible for the tetrameric assembly is mainly mediated by Fn1. In the current model, Ig1 is also in close proximity to the tetrameric interaction region, suggesting it may provide a partial contribution to the assembly, which is supported by our crystallographic observations. The major antiparallel dimer arrangement observed is also the most likely Robo1 entity to exist on the cell surface, with the ECD adopting a compact conformation and orienting the extended Ig1-4 region parallel to the cell surface (Figures 2D and 2E). The flexible linker regions between Ig4-5 and Ig5-Fn1 enable these domains to loop back toward the N terminus before extending down to the cell surface via Fn2-3 (Figure 2E). This positions both the N-terminal Slit2 binding domain (Ig1) and the C-terminal membrane-spanning region in close proximity to the cell surface.

### Cell Surface Imaging of the Robo1 ECD

**Figure 3:**
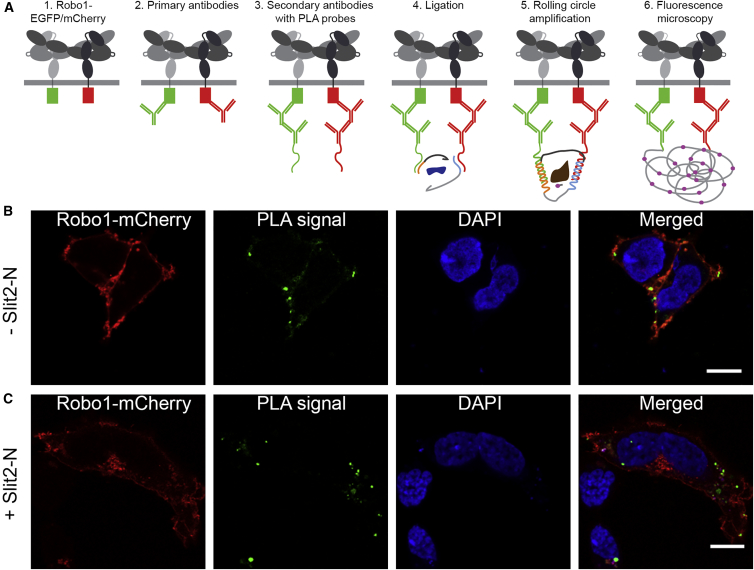
Robo1 Remains Oligomeric on the Cell Surface upon Addition of Slit2-N (A) Schematic overview of the *in situ* fluorescence-based PLA assay used to detect Robo1 oligomerization on the cell surface. (B) An interaction between Robo1-EGFP and -mCherry tagged proteins on the cell surface of HEK293T cells was detected by PLA. (C) This interaction is maintained in the presence of Slit2-N (0.1 μM). Each green dot represents the detection of a Robo1-EGFP and Robo1-mCherry interaction complex, and nuclei were counterstained with DAPI (blue). Single optical slices were acquired with a laser scanning confocal microscope. Robo1-mCherry, PLA interaction, and DAPI are colored red, green and blue, respectively. Scale bars, 10 μm.

To complement our structural work and confirm the presence of Robo1 oligomers on the cell surface, we performed a proximity ligation assay (PLA) as illustrated in Figure 3A (Jarvius et al., 2007). Here, to detect oligomerization of Robo1, cells were co-transfected with Robo1-EGFP and Robo1-mCherry, and PLA was used to detect interactions between EGFP and mCherry. The PLA signal was clearly observed, indicative of Robo1 oligomerization on the cell surface (Figures 3B and S5A). The formation of Robo1-EGFR/Robo1-mCherry dimers is sufficient to generate a PLA signal; however, the nature of this assay does not enable one to determine the number of monomers per oligomer. Furthermore, the presence of a PLA signal upon addition of Slit2-N to cell cultures indicates that oligomers are still present (Figures 3C and S5B). Therefore, a Robo1 monomer to oligomer transition upon addition of Slit2-N does not occur, suggesting another mechanism of receptor activation.

## Discussion

Robo receptors and Slit ligands are well known to play an important role in neuronal and organ development, but after many years it still remains unclear how Slit binding to Robo is translated into downstream signaling. To address this we combined crystallography, SAXS, and 3D EM reconstruction, showing how the Robo1 ECD exists as a large tetrameric assembly *in vitro*. Two symmetrical regions of the EM map, consistent with Robo1 dimer assemblies, can be combined to produce a tetrameric “dimer-of-dimers” configuration (Figures 2D and 2E). Using the Robo1 Ig1-4 fragment and compact nature of the Robo1 Ig1-5 determined by SAXS we could optimally position these fragments in an antiparallel fashion. Subsequently, using symmetry constraints, and adding the Robo1 Fn2-3 structure determined previously (Barak et al., 2014), as well as a model for Fn1, we could build a pseudo atomic model of the whole Robo1 ECD dimer (Figure 2D). We believe this is the most likely Robo1 biological entity to exist on the cell surface because the Robo1 juxtamembrane regions are correctly oriented toward the same cell surface (Figure 2E).

Our observations that Robo1 Ig1-4 and Ig1-5 are monomeric in solution, and others that Fn2-3 is monomeric (Barak et al., 2014), make it difficult to ascertain which region is responsible for dimerization. Our EM-based model predicts that Ig3 is largely responsible, with partial contributions from Ig1 and Ig4, possibly mediated by the crystallographic contact regions identified. The importance of a homophilic and/or heterophilic interaction of the Robo1 and Robo2 extracellular domains was first shown in retinal and olfactory neurite outgrowth assays (Hivert et al., 2002). Later it was reported that Robo1 homophilic binding required the whole ECD (Liu et al., 2004), with this study also revealing the difficulty in identifying the exact interaction region. More recently, the ligand-independent dimerization of *Drosophila* Robo2 was shown to be located within the Ig3-5 region (Evans and Bashaw, 2010), which, together with genetic studies, provides evidence that the diverse response of Robo receptors to Slit may be imparted by structural differences in their extracellular region. The compact nature of the Robo1 ECD we observe supports these studies, where the central Ig5 and Fn1 domains with their longer linker flanking regions facilitate the optimal positioning of the elongated Ig1-4 region for dimerization. In addition, even Robo3, which lost the ability to bind the repellant Slit2 ligand during mammalian evolution (Zelina et al., 2014), has also been shown to interact homophilically (Camurri et al., 2005). It will therefore be important to determine how homo- and heterophilic Robo receptor interactions can influence their individual or collective signaling response to Slit ligands in the future.

**Figure 4:**
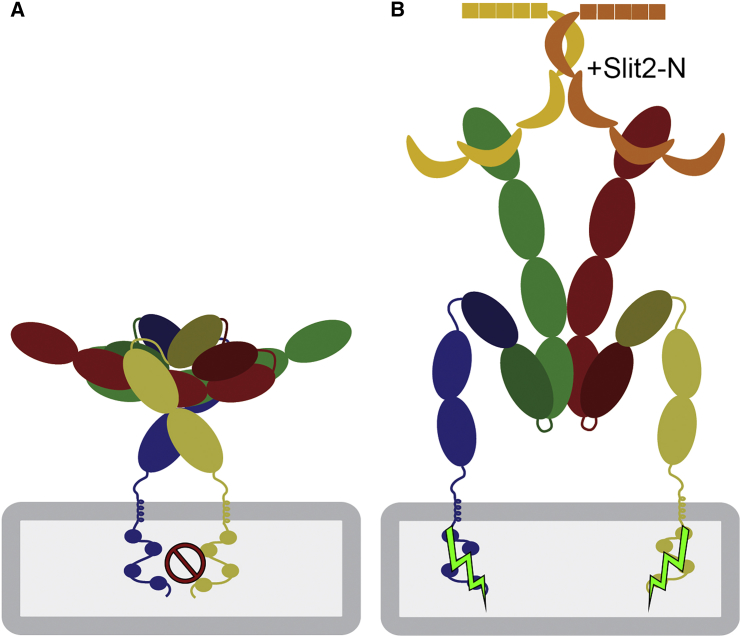
Putative Arrangements of Robo1 on the Cell Surface (A) Compact inactive Robo1 dimers on the cell surface can (B) undergo a conformational change but remain intact upon stimulation with Slit2-N.

The dimer-of-dimers Robo1 ECD arrangement we propose is supported by recent FRET and SpIDA experiments (Zakrys et al., 2014), which report that while both the Ig and Fn repeats contribute to dimerization, the majority is provided by the Ig region. Using an *in situ* PLA assay we could also detect Robo1 oligomers, presumably dimeric, on the cell surface (Figure 3B). Furthermore, a similar PLA signal was observed following incubation with dimeric Slit2-N (Howitt et al., 2004, Seiradake et al., 2009) (Figure 3C), suggesting that Robo1 does not undergo a simple monomer to dimer transition upon Slit-N binding and consistent with previous observations (Zakrys et al., 2014). However, Robo1 dimerization was shown to be important for signaling (Stein and Tessier-Lavigne, 2001). Furthermore, the LRR domains of Slit2 are tethered together by disulfide-bonded linker regions (Howitt et al., 2004), suggestive of an elongated structure incompatible with the compact dimeric assemblies we observe. This allows us to propose a mechanism in which elongated Slit2-N binding to the inactive anti-parallel dimers induces a conformation change in Robo1 dimers that is relayed across the membrane for signaling (Figure 4).

In contrast the tetrameric assembly requires two Robo1 dimers to be oriented in a “back-to-back” configuration, which is reminiscent of a cell-cell contact (Figure 2E). Our model further suggests this cell-cell orientation is mainly mediated by the Fn1 domains, with potential contributions from Ig1. This is consistent with an inactive Robo1 dimer forming a tetrameric assembly with a similar Robo1 dimer on a neighboring cell surface in a back-to-back cell adhesion type manner that may be biologically relevant. Robo2 midline cell expression, observed during the early stages of commissural axon guidance, was shown to have a role in silencing the Slit-Robo1-mediated repulsion of these axons in *Drosophila* (Evans et al., 2015). By co-immunoprecipitation, the deletion of Robo2 Ig1-2 can reduce, but not eliminate, an interaction with Robo1, which suggests other regions also contribute. While further experiments are clearly required, these studies strongly suggest that a *trans* Robo1-Robo2 receptor interaction is important during axonal guidance by preventing Slit binding to Robo1. Our tetrameric assembly provides a potential insight into how this may occur, whereby compact and inactive Robo1 dimers on the approaching axonal cell surface could be further stabilized by Robo2 dimers on midline cells in a back-to-back dimeric configuration to attenuate their response to Slit ligands. Such a tetrameric assembly on the cell surface can be disrupted as Slit2 is introduced, leading to a weakening of the cell-cell adhesion.

In conclusion, our results strongly support a mechanism in which inactive Robo1 dimers undergo a conformational change upon binding Slit2-N (Figure 4). In fact this mechanism is reminiscent of PlexinA4 activation by Sema6A, in that semaphorin disrupts an inhibitory plexin dimer to induce an active dimer (Kong et al., 2016, Marita et al., 2015). This Robo1 conformation change could be further enhanced by cell surface heparan sulfate (Hu, 2001, Hussain et al., 2006) and promote the recruitment of effector proteins, such as SOS, for cellular signaling (Chance and Bashaw, 2015). As such our results could be exploited for the development of new therapeutics by screening for Robo1 receptor antagonists stabilizing an inactive conformation on the cell surface.

## STAR★Methods

### Key Resources Table

**Table:** 

REAGENT or RESOURCE	SOURCE	IDENTIFIER
**Antibodies**		

Living Colors® A.v. Monoclonal Antibody (JL-8)	Clontech	Cat. #632381; RRID: AB_2313808
Living Colors® DsRed Polyclonal Antibody	Clontech	Cat. #632496; RRID: AB_10013483
Anti Influenza A nucleoprotein mouse monoclonal	Argene	Ref. 11-030

**Chemicals, Peptides, and Recombinant Proteins**		

Slit2-N	R&D systems	cat. #5444-SL-050

**Critical Commercial Assays**		

DuoLink In Situ Fluorescence	Sigma	DUO92101-1KT

**Deposited Data**		

Crystal structure of Robo1 Ig1-4 (1)	This study	PDB: 5o5g
Diffraction images for Robo1 Ig1-4 (1)	This study	https://doi.org/10.15785/SBGRID/503
Crystal structure of Robo1 Ig1-4 (2)	This study	PDB: 5ope
Diffraction images for Robo1 Ig1-4 (2)	This study	https://doi.org/10.15785/SBGRID/504
Crystal structure of Robo1 Ig5	This study	PDB: 5o5i
Diffraction images for Robo1 Ig5	This study	https://doi.org/10.15785/SBGRID/505

**Experimental Models: Cell Lines**		

HEK-293T cell line	ATCC	ATCC CRL-3216

**Oligonucleotides**		

5’-CAGCACTAGGGATCCCTTCGTCAGGAAG ATTTTCCA-3’	This study	N/A
5’-TCGTCGATCAGCGGCCGCTGTAACTTCC AAATATGC-3’	This study	N/A
5’-TCGTCGATCAGCGGCCGCTTGAACTTCA ATGTAAGC-3’	This study	N/A
5’-TCGTCGATCAGCGGCCGCTGCCACTTC CACACT-3’	This study	N/A

**Recombinant DNA**		

Plasmid: pTT3_Robo1_ECDa	This study	N/A
Plasmid: pTT3_Robo1_ECDb	This study	N/A
Plasmid: pTT3_Robo1_Ig1-4	This study	N/A
Plasmid: pTT3_Robo1_Ig1-5	This study	N/A
Plasmid: pHLSec_Ig5	This study	N/A
Plasmid: pmCherry-N1_Robo1	This study	N/A
Plasmid: pmEGFP-N1_Robo1	This study	N/A
Plasmid: Influenza A NP-mCherry (for PLA control)	(Avilov et al., 2016)	N/A

**Software and Algorithms**		

XDS	(Kabsch, 2010)	http://xds.mpimf-heidelberg.mpg.de
Phaser	(McCoy et al., 2007)	http://www.phaser.cimr.cam.ac.uk
Coot	(Emsley et al., 2010)	http://www2.mrc-lmb.cam.ac.uk/personal/pemsley/coot
Buster	(Bricogne et al., 2016)	http://www.globalphasing.com/buster
MolProbity	(Chen et al., 2010)	http://molprobity.biochem.duke.edu
PyMol	Molecular Graphics System, Version 1.8.6 Schrodinger, LLC	http://www.pymol.org
PRIMUS	(Konarev et al., 2003)	http://www.embl-hamburg.de/biosaxs/primus.html
GNOM	(Svergun, 1992)	http://www.embl-hamburg.de/biosaxs/gnom.html
DAMMIF	(Franke and Svergun, 2009)	http://www.embl-hamburg.de/biosaxs/dammif.html
DAMAVER	(Volkov and Svergun, 2003)	http://www.embl-hamburg.de/biosaxs/damaver.html
SUPCOMB	(Kozin and Svergun, 2001)	http://www.embl-hamburg.de/biosaxs.supcomb.html
CRYSOL	(Svergun et al., 1995)	http://www.embl-hamburg.de/biosaxs/crysol.html
CORAL	(Petoukhov et al., 2012)	http://www.embl-hamburg.de/biosaxs/coral.html
EMAN	(Ludtke et al., 1999)	http://blake.bcm.edu/emanwiki/EMAN2
CTFFIND3	(Mindell and Grigorieff, 2003)	http://grigoriefflab.janelia.org/ctf
Bsoft	(Heymann, 2001)	http://lsbr.niams.nih.gov/bsoft
IMAGIC-5	(van Heel et al., 1996)	http://www.imagescience.de/imagic.html
VIPER	(Penczek, 2014)	http://sphire.mpg.de
RELION	(Scheres, 2012)	http://www2.mrc-lmb.cam.ac.uk/relion
Chimera	(Pettersen et al., 2004)	http://www.cgl.ucsf.edu/chimera
iMODFIT	(Lopez-Blanco and Chacon, 2013)	http://chaconlab.org/hybridem/imodfit
Consurf	(Ashkenazy et al., 2016)	http://consurf.tau.ac.il/2016
EPPIC	(Duarte et al., 2012)	http://www.eppic-web.org/ewui/
SBgrid	(Morin et al., 2013)	http://sbgrid.org

**Other**		

QuixStand Hollow Fibre	GE HealthCare Life Sciences	Prod: #56-4101-81
Ni-sepharose FastFlow resin	GE HealthCare Life Sciences	Prod: #17-5318-02
HiLoad 16/60 Superdex 200 column	GE HealthCare Life Sciences	Prod: #28989335
Superdex 200 Increase 10/300 GL	GE HealthCare Life Sciences	Prod: #28990944

### Contact for Reagent and Resource Sharing

Further information and requests for reagents should be directed to, and will be fulfilled by the Lead Author, Dr. Andrew A. McCarthy (andrewmc@embl.fr).

### Method Details

#### Production and Purification of Robo1 Protein Constructs

Robo1 constructs spanning residues 454-543 (Robo1 Ig5), 61-446 (Robo1 Ig1-4), 61-543 (Robo1 Ig1-5), 61-853 (Robo1 ECDa), 61-864 (Robo1 ECDb) were produced by PCR amplification using the full-length cDNA coding for human Robo1 (NP_002932) (Figure S1A). Robo1 Ig1-4, Robo1 Ig1-5, Robo1 ECDa and ECDb were cloned into a modified pTT3 expression vector (Durocher et al., 2002) encoding a signal peptide and a C-terminal hexa-histidine tag. Robo1 Ig5 was cloned into the pHLSec expression vector (Aricescu et al., 2006). All proteins were produced by transient expression in Human Embryonic Kidney cells (HEK-293T) as previously described (Morlot et al., 2007). Expression media containing the various Robo1 constructs were harvested 6 days after transfection. Cells were separated from the expression media with a QuixStand Hollow Fibre System (GE Healthcare Life Sciences) using a cartridge with a 0.2 μM molecular size cut off. The expression media were subsequently concentrated 10-fold and diafiltrated (200 mM NaCl and 20 mM Tris pH=7.5) using the same Quixstand system with a 10 kDa cut off cartridge. The Robo1 constructs were purified from the concentrated media by the batch binding method to Ni-sepharose FastFlow resin (GE Healthcare Life Sciences) charged with Ni^2+^. This resin was subsequently washed with buffer containing 500 mM NaCl and 20 mM Tris pH=7.5. Non-specifically bound proteins were eluted with 25 mM imidazole in the same buffer. The purified proteins were subsequently eluted using an imidazole gradient up to 500 mM imidazole and analysed by SDS-PAGE. Fractions containing the relevant protein were pooled and further purified by size-exclusion chromatography (Superdex S200, GE Healthcare Life Sciences) in 200 mM NaCl and 20 mM Tris pH 7.5 (Figure S1B). Proteins used for Gradient Fixation (GraFix) were eluted in 200 mM NaCl and 20 mM Hepes pH 7.5.

#### Multiangle Light Scattering (MALS)

A Hitachi HPLC and Elite LaChrom UV detector L-2400 coupled to a Wyatt Dawn HELEOS-II multi-angle static light scattering detector and Optilab-T-rex refractometer were used for SEC-MALS. A Superdex S200 HR 10/30 size exclusion column (GE Healthcare Life Sciences) was equilibrated in a running buffer consisting of 200 mM NaCl and 20 mM Tris pH 7.5. A 55 μl sample volume of Robo1 ECDa at 2.5 mgs/ml originating from peak 1 fractions in Figure S1B was used, and the data were analyzed using ASTRA (Wyatt Technology) with a dn/dc value of 0.185 (Figure S1C).

#### Crystallization and Data Collection

Crystals of Robo1 Ig1-4 were grown in hanging drops at 20°C using solutions containing 6% PEG 4000, 100 mM Na citrate pH 4.0, and 10 mM CaCl_2_ for crystal form 1 or 0.5 M ammonium phosphate, and 100 mM Na citrate pH 5.6 for crystal form 2. Crystals of Robo1 Ig5 grew from hanging drops at 20°C using solutions containing 100 mM Na acetate pH 4.5 and 25% PEG 3350. All crystals were flash frozen at 100 K after transferring them to identical crystallization conditions containing 20% glycerol. Robo1 Ig1-4 crystallized in space group C222_1_ and contained one molecule in the asymmetric unit. The only difference between the two crystal forms is an elongation of the C-axis by ∼10Å (Table 1). Robo1 Ig5 crystallized in space group P6_1_ and also contained one molecule in the symmetric unit. All data was collected on ID14-4 (McCarthy et al., 2009) at the European Synchrotron Radiation Facility (ESRF), and integrated using the XDS suite (Kabsch, 2010). A summary of the data statistics is given in Table 1.

#### Structure Determination and Refinement

Robo1 Ig1-4 crystal form 1 was solved by molecular replacement using Robo1 Ig1-2 (Morlot et al., 2007) as a search model with Phaser (McCoy et al., 2007) and crystal form 2 was solved with Phaser (McCoy et al., 2007) using Robo1 Ig1-4 crystal form 1. Robo1 Ig5 was also solved by molecular replacement with phaser (McCoy et al., 2007) with a sequence homology model generated using the Robo1 Ig1 and Ig2 domains as a template. Several rounds of manual building with Coot (Emsley et al., 2010), and structure refinement with BUSTER (Bricogne et al., 2016) were carried out for all structures. MOLPROBITY (Chen et al., 2010) was used for model validation and all the crystallographic information is summarized in Table 1. Structural figures were prepared with PyMOL (Schrödinger, LLC).

#### Small Angle X-ray Scattering (SAXS)

SAXS measurements were performed at the EMBL X33 beamline (DESY, Hamburg) (Roessle et al., 2007) using a MAR345 area detector. The sample–detector distance was 2.7 m. Data were collected at concentrations of 1 to 4 mg/mL with exposure times of 120 s at 10°C. SAXS intensity *I(s)* is represented as a function of the momentum transfer modulus *s=4π sin(θ)/λ*, where *λ* is the radiation wavelength (0.15 nm), and *2θ* is the scattering angle. Scattering from the buffer was collected before and after that of the sample, then averaged and subtracted from the sample using the program PRIMUS (Konarev et al., 2003). To avoid the aggregation effect, the data recorded at the lowest concentrations were used in further analysis. The radii of gyration *R*_*g*_ were evaluated using the Guinier approximation (Guinier, 1939), assuming that at very small angles (*s<1.3/R*_*g*_) the intensity is represented as *I(s)=I(0) exp(–1/3(sR*_*g*_*)*^*2*^*)* and the volumes of the hydrated particles, *V*_*p*_, were estimated using the Porod invariant (Porod, 1982). The maximum dimensions *D*_*max*_ were computed using the indirect transform package GNOM (Svergun, 1992), which also provides the distance distribution functions *p(r)*. Multiple independent *ab initio* reconstructions of low resolution three-dimensional shapes with the best fit to the experimental scattering curves were done using the program DAMMIF (Franke and Svergun, 2009) and subsequently aligned and averaged in the program DAMAVER (Volkov and Svergun, 2003). The averaged low-resolution SAXS shapes were aligned with the Robo1 Ig1-4 models using the program SUPCOMB (Kozin and Svergun, 2001), which minimizes normalized spatial deviation (NSD) between the models. The scattering from the atomic models was calculated using the program CRYSOL (Svergun et al., 1995). Rigid body modelling of Robo1 Ig1-4 and Robo1 Ig5 with addition of the missing interdomain loops was performed by the program CORAL (Petoukhov et al., 2012).

#### Negative Stain EM Reconstruction

Preliminary negative stain EM imaging of Robo1 ECDa after gel filtration indicated that Robo1 formed globular oligomers but also smaller elongated species. Thus, we used a Gradient Fixation (GraFix) protocol (Kastner et al., 2008) to stabilize the oligomers and separate them from the unassembled background. Robo1 ECDa samples were subjected to ultracentrifugation for 18 hrs at 4°C in a 10-30% glycerol gradient (with the 30% glycerol solution containing 0.15% glutaraldehyde) using a SW60Ti rotor (Beckman Coulter). The gradient tubes were fractionated into 60 μl fractions and analysed by SDS-PAGE (7.5%) (Figures S1D and S1E). The most homogeneous fractions were selected and immediately applied to the clean side of a thin carbon on a carbon-mica interface. The carbon film with the adsorbed sample was floated on a drop of 2% (weight per volume) ammonium molybdate solution. A 400-mesh copper grid was put on top of the floating carbon film, and the whole setup turned upside down to catch a second layer of carbon floating on another drop of ammonium molybdate. Prepared this way, trapped between two layers of carbon, the sample was uniformly stained.

Images were recorded under low-dose conditions with a JEOL 1200-EX II microscope at 100 kV on photographic film using a nominal magnification of 40,000x. The best fraction, in terms of homogeneity of particles on the EM grid, was number 12 (Figure S1D), from which a total of 75 negatives were collected. These negatives were digitized on a Zeiss scanner (Photoscan TD) to a pixel size of 3.5 Å at the object scale (Figure 2A). A semi-automatic particle selection with the EMAN boxer routine (Ludtke et al., 1999) led to an extraction of a total of ∼68,500 subframes of 80x80 pixels that were CTF-corrected with CTFFIND3 (Mindell and Grigorieff, 2003) and Bsoft (Heymann, 2001), and low-path filtered at 15 Å. This data set was subjected to multivariate statistical analysis and classification with IMAGIC-5 (van Heel et al., 1996), which led to a removal of ∼11% of the images. The resulting 2D class averages suggested a C2 or D2 particle symmetry (Figure 2B). Three different sets of 20 to 30 best class averages were extracted and used to calculate 12 *ab initio* 3D reconstructions of Robo1 ECDa with VIPER (Cheng et al., 2015, Penczek, 2014), applying either a C2 or a D2-symmetry. Even when a C2-symmetry was used for calculations, the resulting 3D reconstructions appeared D2-symmetrical. Moreover, all plausible solutions had a very similar architecture with a well-defined elongated and “kinked’ subunit shape. Therefore, they were aligned in 3D, averaged, D2-symmetrized, and filtered to 60 Å resolution to create a reliable initial model of Robo1 ECDa. This model was further refined in RELION (Scheres, 2012) using 3D auto-refinement and classification procedures. 3D classification into four classes that looked alike and contained a similar number of particles, and a fourth minor class that we attributed to broken or misaligned particles. Particles from the three major classes, representing ∼86% of the input images, were grouped together and refined in 3D as a single class of 53,702 particles (Figure S4A), resulting in the final 3D negative stain EM map (Figures 2D, S4A, and S4B) with a gold-standard resolution of 16 Å according to the FSC=0.143 criterion (Figure 2C). This final map was filtered to 20 Å resolution for model building (Figures 2D and S4B). Later, when analyzing the Robo1 Fn2-3 structure (Barak et al., 2014) we discovered that the Robo1 ECDa construct was missing the last β-strand of Fn3. We therefore made a second construct encompassing the whole ectodomain (Robo1 ECDb) and verified that its negative stain EM appearance was indistinguishable from Robo1 ECDa.

An initial model of Robo1 ECDa was obtained by manually positioning the crystal structures of Robo1 Ig1-4 and Ig5 (Figures 1B and 1D), the extracellular juxtamembrane region previously determined [PDB: 4hlj (Barak et al., 2014)], and a molecular model of the first fibronectin domain (Fn1) into the EM density using Chimera (Pettersen et al., 2004). The domains were placed into the map by imposing some restraints, including the D2 symmetry, the extended form of Robo1 Ig1-4 (Figure 1B), and the low resolution Robo1 Ig1-5 SAXS model (Figure S3B). With these restraints, the manually positioned Robo1 ECDa was then fitted as one rigid body using the ‘fit-in-map’ module of Chimera. Following this, the density corresponding to a Robo1 monomer was extracted, and flexible fitting was performed using iMODFIT (Lopez-Blanco and Chacon, 2013). For the flexible fitting, all individual domains were fixed, and only the linker regions: Ig1-Ig2, Ig4-Ig5, Ig5-Fn1, and Fn1-Fn2 were allowed to move. This monomeric model (Figure S4B) was then docked into the whole EM map and symmetry-related molecules were generated using Chimera (Figure 2D). A comparison of 20Å filtered projections generated from the final EM map and model is supportive of our fitting (Figure S4C). All EM figures were prepared with Chimera.

#### Proximity Ligation Assay

Full length Robo1 (residues 1-1651) was cloned into pmEGFP-N1 and pmCherry-N1 (Clontech). HEK-293T cells were co-transfected with Robo1-EGFP and Robo1-mCherry constructs using PEI transfection reagent as described previously (Aricescu et al., 2006); 1-2 days post-transfection, the cells were treated with 0.1 μM mouse Slit2-N (R&D systems, cat. #5444-SL-050) and (Figure S1F), or PBS, and fixed 30 min after adding Slit2-N, using 4% PFA in PBS for 20 min at room temperature. After fixation, the cells were permeabilised with 0.1% Triton X100 in PBS for 10 min, blocked with 3% normal rabbit serum for 60 min, and then incubated with a mixture of anti-GFP monoclonal antibody JL-8 (Clontech) diluted 1:50, and rabbit Living Colors® anti-DsRed polyclonal antibody, which recognizes mCherry (Clontech, Cat. #632496), diluted 1:100, in the presence of 3% normal rabbit serum. For negative and positive controls (Figures S5C and S5D), mouse monoclonal antibody against the nucleoprotein (NP) of influenza A virus (Argene, ref. 11-030) diluted 1:200 was used. Further, to detect *in situ* if the epitopes are within 40 nm proximity (Soderberg et al., 2006), cells were subjected to proximity ligation assay (PLA) with DuoLink II fluorescence kit (Sigma) according to the manufacturer’s protocol. Anti-mouse PLA probe MINUS, anti-rabbit PLA probe PLUS and Detection Reagents Far Red were used. As assay controls we used cells transfected with two independent plasmids encoding NP and mCherry respectively (for negative control) or an influenza A NP-mCherry tagged protein (for positive control) (Avilov et al., 2016). For technical negative controls, samples with omitted primary antibodies were used. Images were acquired with LSM780 confocal laser scanning microscope (Carl Zeiss) with 63x 1.4 N.A. oil immersion objective; excitation and emission, respectively, were set at 561 nm and 590-670 nm for mCherry; 633 nm and 643-742 nm for PLA signal (Far Red detection reagent); 405 nm and 420-470 nm for DAPI. Signals were acquired sequentially and images were corrected for cross-talk between the channels.

### Data and Software Availability

The atomic coordinates and structure factors for the crystal structures presented in this study have been deposited in the Protein Data Bank under ID codes 5o5g and 5ope (Robo1 Ig1-4 crystal form 1 and 2 respectively), and 5o5i (Robo1 Ig5).

## Supplemental Information

Document S1. Figures S1–S5 and Table S1

Document S2. Article plus Supplemental Information
