# Supplementary material for: Robo1 Forms a Compact Dimer-of-Dimers Assembly
Source: Structure. 2018 Feb 6;26(2):320–328.e4. doi: 10.1016/j.str.2017.12.003 (PMC5807052; doi:10.1016/j.str.2017.12.003)
Supplement: Document S1. Figures S1–S5 and Table S1 [file mmc1.pdf]

Structure, Volume *26*

Supplemental Information

Robo1 Forms a Compact Dimer-of-Dimers Assembly

Nataliia Aleksandrova, Irina Gutsche, Eaazhisai Kandiah, Sergiy V. Avilov, Maxim V. Petoukhov, Elena Seiradake, and Andrew A. McCarthy

Supplementary information for

Robo1 forms a compact dimer of dimers assembly

Nataliia Aleksandrova[1], Irina Gutsche[2], Eaazhisai Kandiah[2], Sergiy V. Avilov[1], Maxim V. Petoukhov[3,4,5], Elena Seiradake[6] and Andrew A. McCarthy[1]

[1]*European Molecular Biology Laboratory, Grenoble Outstation, 71 avenue des Martyrs, F-38042 Grenoble, France*.

[2]*University Grenoble Alpes, CNRS, CEA, IBS, 71 avenue des Martyrs F-38044 Grenoble, France*.

[3]*European Molecular Biology Laboratory, Hamburg Unit, EMBL c/o DESY, Hamburg 22607, Germany*.

[4]*Federal Scientific Research Centre 'Crystallography and Photonics' of Russian Academy of Sciences, Leninsky prospect 59, 119333 Moscow, Russian Federation.*

[5]*A. N. Frumkin Institute of Physical Chemistry and Electrochemistry RAS, Leninsky prospect 31, 119071 Moscow, and N.N. Semenov Institute of Chemical Physics of Russian Academy of Sciences, Kosygina street 4, 119991 Moscow, Russian Federation*.

[6]*Department of Biochemistry, University of Oxford, South Parks Road, Oxford OX1 3QU, Oxford, United Kingdom*.

Correspondence should be addressed to A. A. McC. *e-mail:* andrewmc@embl.fr (Phone: +33-476207276 fax: +33-476207199)

Correspondence author contact details:

Phone: +33-476207276

fax: +33-476207199

e-mail: andrewmc@embl.fr

SUPPLEMENTAL FIGURES

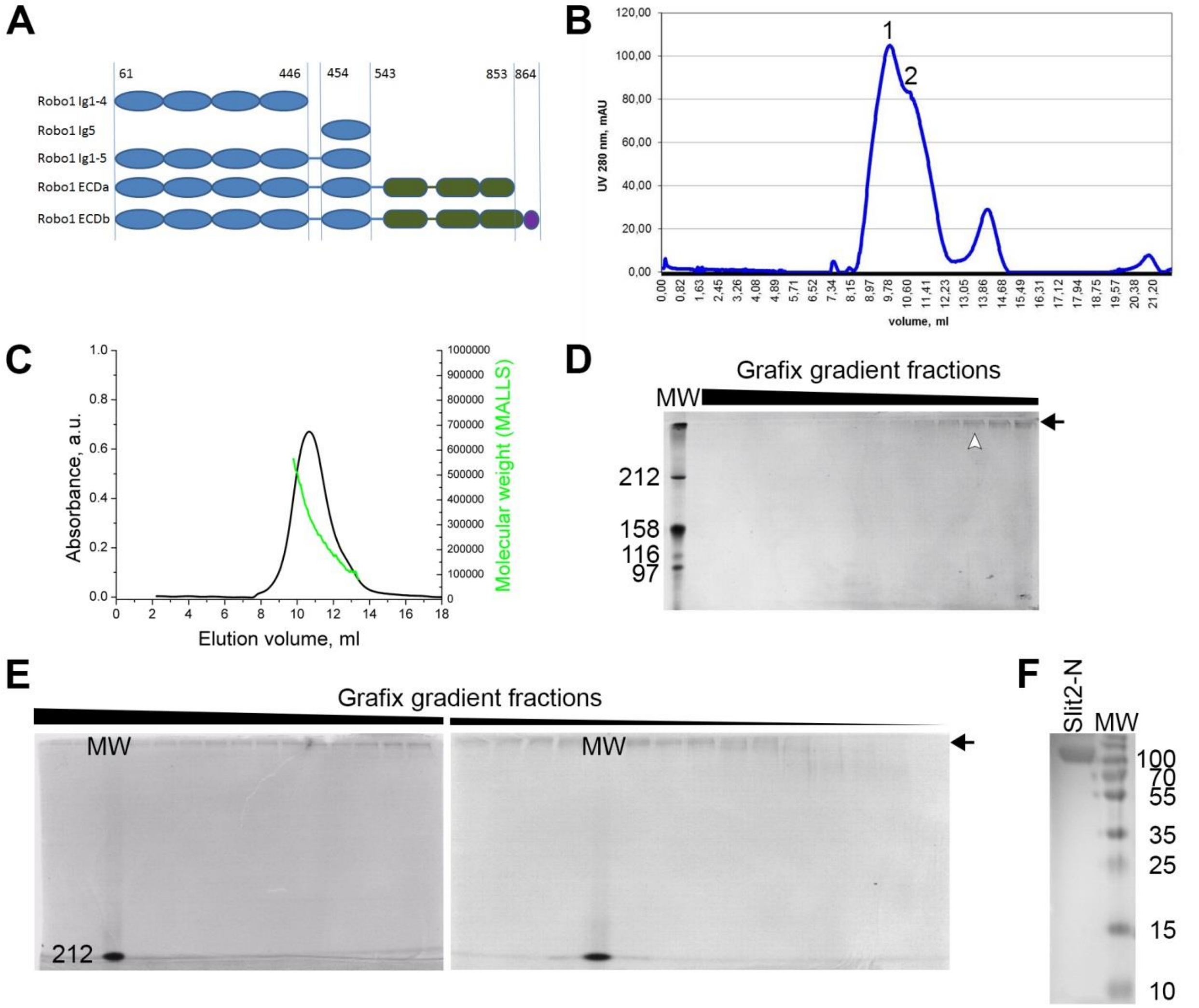

Figure S1. Related to Figure 1, 2 and 3. Robo1 ECD predominantly forms tetramers *in vitro* (A) Robo1 domain architecture and expression constructs used in this study. (B) Size exclusion chromatogram of Robo1 ECDa showing tetrameric (1) and dimeric (2) species. (C) Size exclusion chromatography and multi angle light scattering (MALS) of the Robo1 ECDa peak 1 fraction from (B). The absorbance at 280 nm and molecular weight deduced from light scattering data are shown. (D) SDS PAGE (7.5 %) gel from a standard (50 minutes) run of the first 14 glutaraldehyde gradient fixation (GraFix) cross-linking fractions used to determine those for negative stain EM grid preparation. Fraction number 12, marked with a white arrow head, was eventually chosen. Molecular weights (MW) are indicated and gradient direction is shown as a triangle, revealing a primarily tetrameric Robo1 (marked by arrows). (E) An extended (90 minute) SDS PAGE (7.5 %) of the first 28 GraFix fractions was also run to try and improve the gel quality and confirm the Robo1 MW was much greater than 212 kDa, the largest MW marker available. The later fractions showed extensive smearing and were not analyzed further. (F) A representative SDS PAGE (15 %) gel of Slit2-N used for proximity ligation assays.

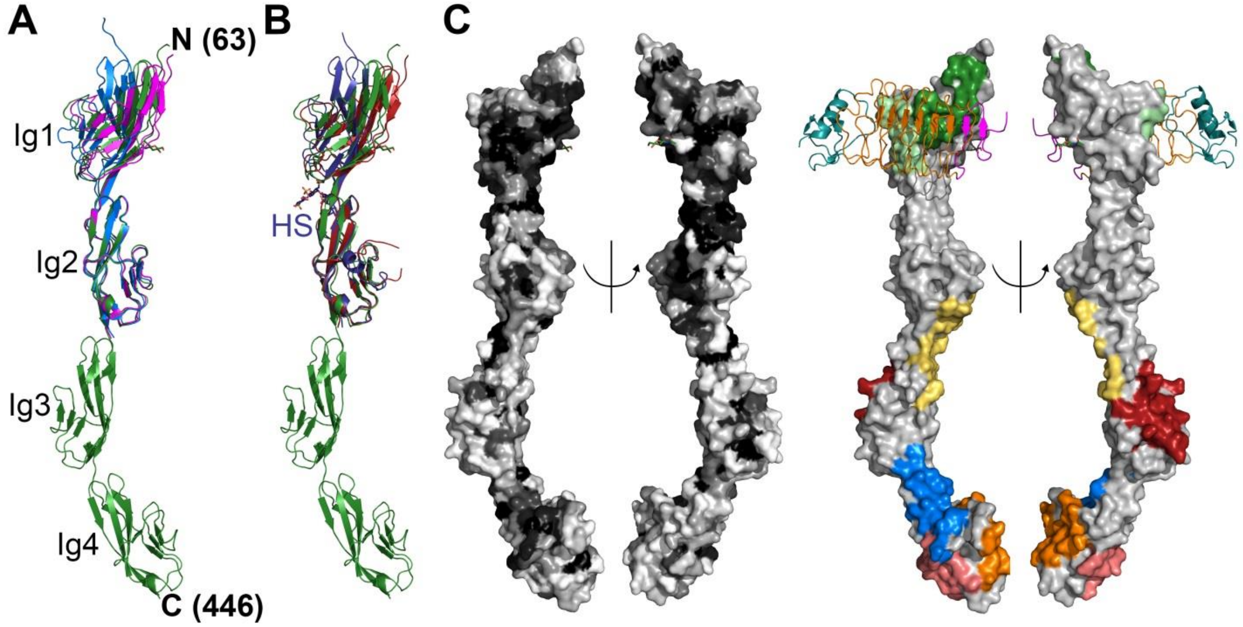

Figure S2. Related to Figure 1. Structural comparison of human Robo1 Ig1-4 to Ig1-2 and *Drosophila* (d)Robo1 Ig1-2 structures, and Robo1 Ig1-4 sequence conservation (A) The Ig1-2 domains in Ig1-4 [PDB: 5o5g (green)] are most similar to the Robo1 Ig1-2 conformation 2 [PDB: 2v9r (magenta)], Robo1 Ig1-2 conformation 1 [PDB: 2v9q] is shown in marine. The N-glycosylation at Asn160 is shown in stick representation. (B) The Ig1-2 domains in Ig1-4 [PDB: 5o5g (green)] adopt a similar conformation to the apo dRobo1 Ig1-2 [PDB: 2vr9 (red)], dRobo1 Ig1-2 bound to heparan sulphate (HS), shown in stick representation [PDB: 2vra], is coloured blue. (**C**) Sequence conservation among Robo receptors mapped on to Robo1 Ig1-4 using the Consurf server and coloured from black (highly conserved) to white (not conserved). For comparison Figure 2C highlighting the crystallographic contacts mediated by Ig1, Ig3 and Ig4 is shown on the right. Interface 1 is symmetric and mediated by Ig4 (blue); interface two is mediated by Ig2-Ig3 (yellow) and Ig4 (orange); and interface three is mediated by Ig3 (red) and Ig4 (salmon); interface four is mediated by Ig1 (light and dark green) and overlaps the Slit2 D2 cyan binding site, illustrated as a ribbon representation (N and C-terminal caps coloured in magenta and cyan respective and LRR coloured in orange.

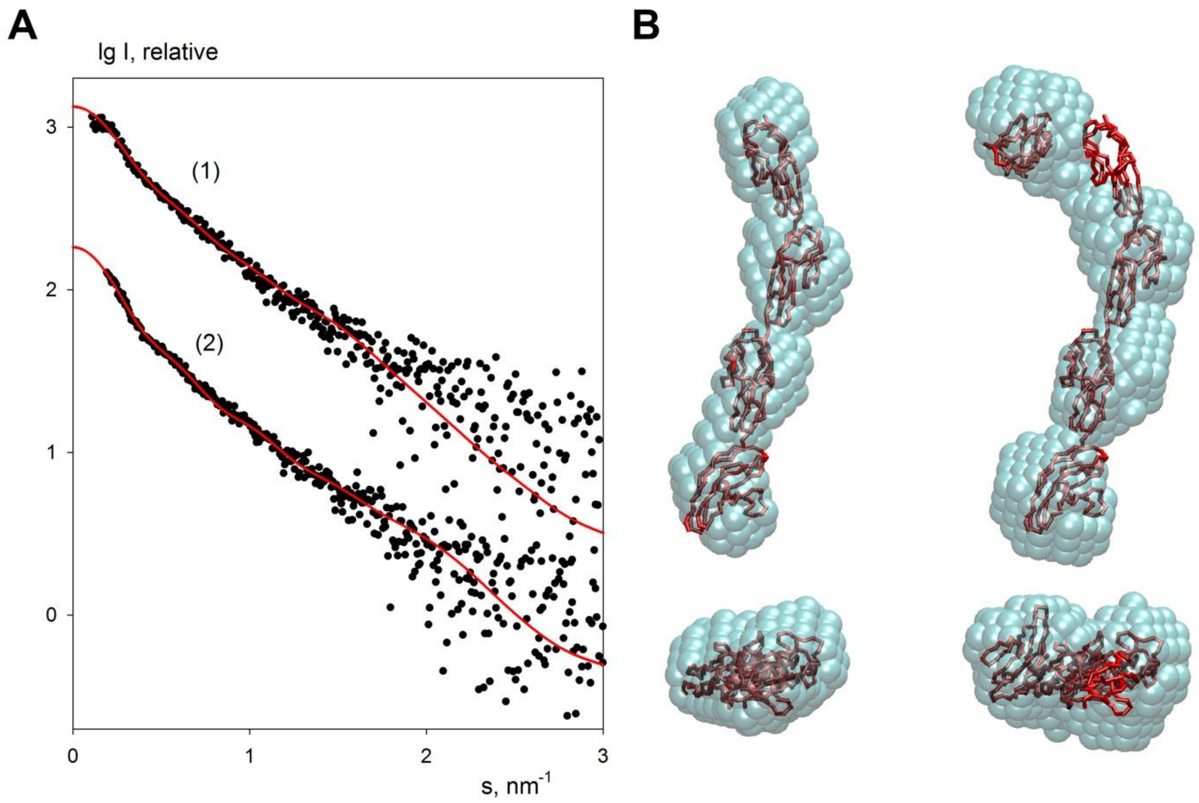

Figure S3. Related to Figure 2. Small-angle X-ray scattering (SAXS) of Robo1 Ig1-4 and Robo1 Ig1-5 (A) SAXS patterns of Robo1 Ig1-4 (1) and Robo1 Ig1-5 (2). Experimental data are denoted by black dots and the fits computed from the atomic model of Robo1 Ig1-4 and the rigid body model of Robo1 Ig1-5 are given as red solid lines. The curves are displayed in logarithmic scale for better visualization. (B) Structural modelling of Robo1 Ig1-4 (left) and Robo1 Ig1-5 (right) *ab initio* shapes are presented as cyan beads. The atomic model of Robo1 Ig1-4 and the typical rigid body model of Robo1 Ig1-5 are presented as red backbone traces. Bottom view is rotated by 90° about the horizontal axis.

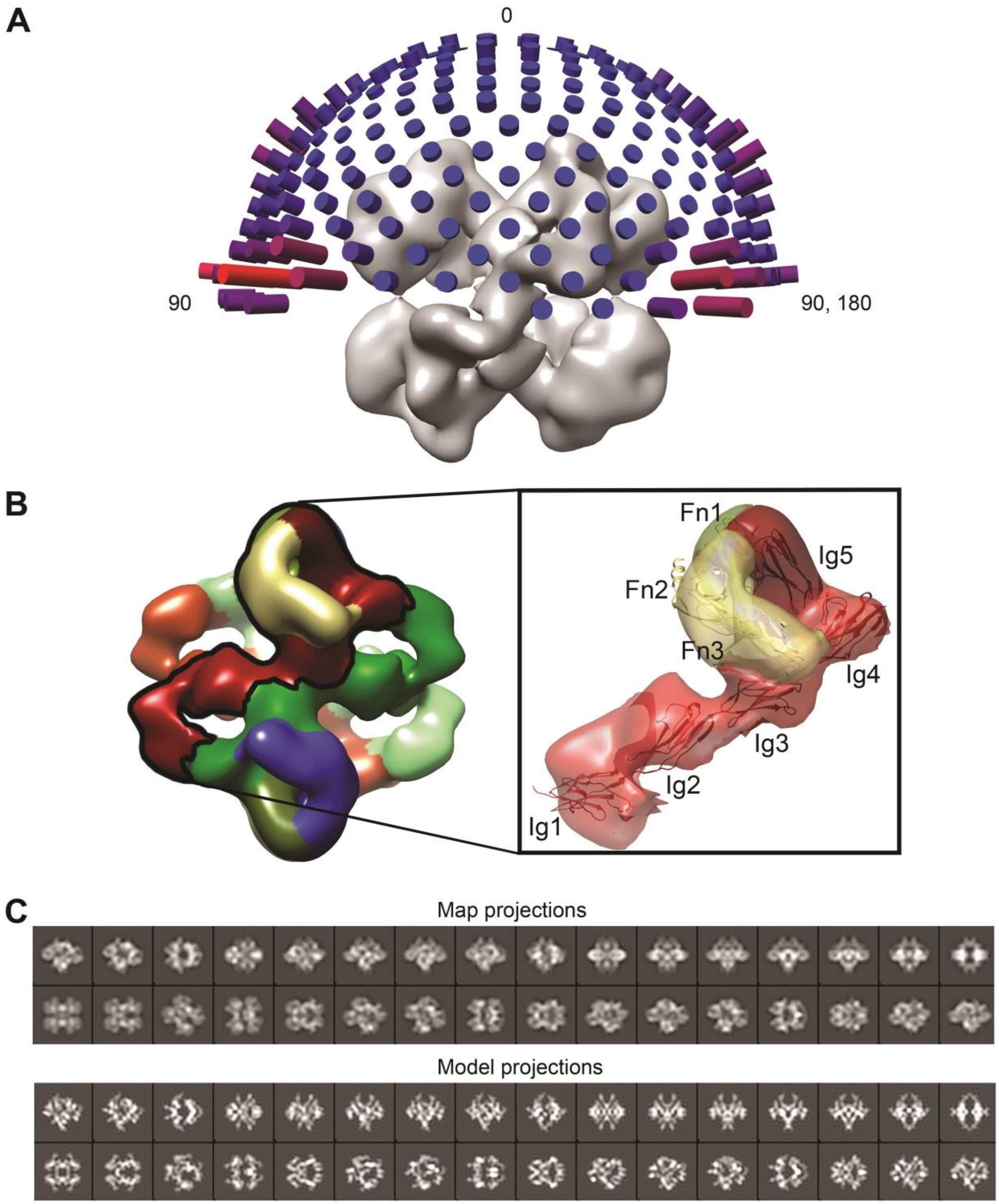

Figure S4. Related to Figure 2. EM reconstruction of the Robo1 ectodomain (A) Distribution of Euler angles overlaid on the refined map to facilitate visualization of the corresponding relative orientation of the particles. (B) Segmentation of the 3D reconstruction (left) to facilitate visualisation and domain assignment of a Robo1 ectodomain monomer (right). The Ig and Fn domains are labelled and segmentation is coloured as in Figure 2D. (C) Comparison between projections generated using the final 3D EM map and a ~20 Å filtered 3D volume calculated from the Robo1 ECD model.

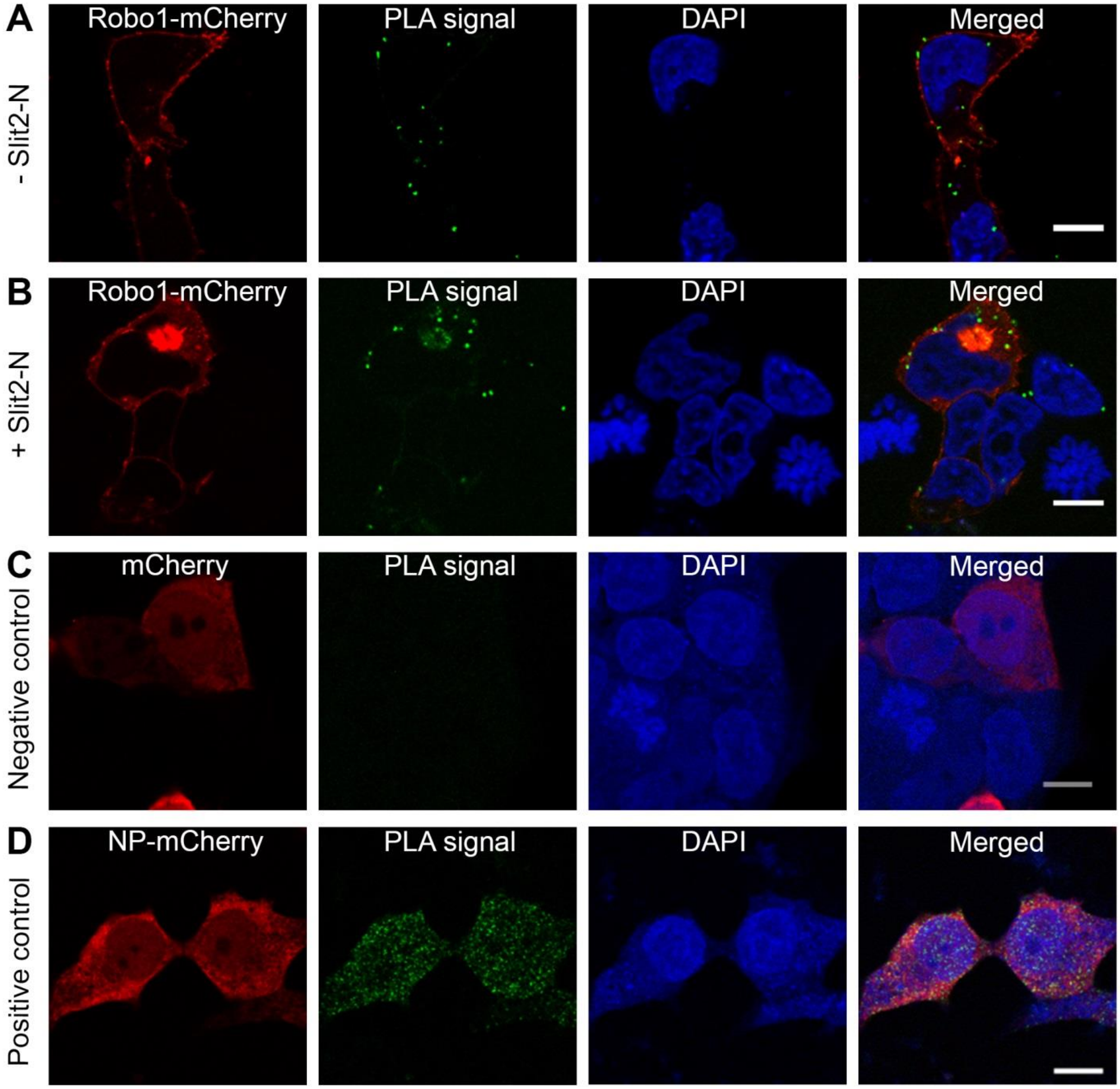

Figure S5. Related to Figure 3. Robo1 remains oligomeric on the cell surface upon addition of Slit2-N. An interaction between Robo1-EGFP and -mCherry tagged proteins on the cell surface of HEK-293T cells was detected in the absence (A) or presence (B) of Slit2-N (0.1 μM). (C) Negative control of cells expressing nucleoprotein (NP) and mCherry proteins from two separate plasmids. (D) Positive control of cells expressing a NP-mCherry fusion labelled protein with antibodies against NP and mCherry. Each green dot represents the detection of a Robo1-EGFP and Robo1-mCherry interaction complex or NP-mCherry fusion protein in the positive control, with nuclei counterstained using DAPI. Single optical slices were acquired with a laser scanning confocal microscope. mCherry, PLA-signal, and DAPI are pseudo-coloured red, green and blue respectively. The scale bar is 10 μm.

SUPPLEMENTAL TABLE

Table S1. Overall Results obtained by SAXS. Related to Figure 2.

Protein	Concentration, mg/ml	R_g, nm	D_{max}, nm	V_p, nm^3	χ^2
Robo1 Ig1-4	1.1	4.5±0.2	18.5±0.5	95	1.4
Robo1 Ig1-5	1.4	4.9±0.3	20.0±0.5	135	1.3
